# Injury-related risk behaviors among secondary technical vocational school students in China: a cross-sectional survey

**Published:** 2019-08

**Authors:** Fenfen Li, Shumei Wang

**Affiliations:** ^*a*^Key Laboratory of Public Health Safety, Ministry of Education-Department of Children and Adolescent, Health, School of Public Health, Fudan University, Shanghai (200032), China.

## Abstract

**Background:**

To understand the prevalence of daily injury-related risk behaviors among secondary technical vocational school students, and to provide scientific proof and effective guidance for further injury intervention.

**Methods:**

A cluster sampling method was used to conduct a questionnaire survey of all grade one and grade two students in a secondary technical vocational school in Jiangsu Province in April 2018, to understand the current status of injury-related risk behaviors.

**Results:**

Secondary technical vocational school students traffic risk behaviors, bullying and violence experience, sports risk behaviors, unsafe animal contact, were highly prevalent. The following behaviors occurred more than 25%: running a red light, not using the crosswalk, playing electronic equipment while crossing the road, not using a helmet, not wearing a seat-belt, not using reflective equipment at night, no preparation activities before sports, no protective measures during exercise, playing with stray cats, etc.

**Conclusions:**

Injury-related risk behaviors among secondary technical vocational school students were highly prevalent. The intervention of such risk behaviors should aim at characteristics of the population and their behaviors, take full advantage of families, schools, communities and other resources to solve intervention obstacles and promote correct behavior recognized as social norms, and create safe environment and atmosphere of families, schools, communities, and the whole society.

**Keywords:**

Secondary technical vocational school, Injury, Risk behavior

